# Supplementary material for: Mixed evidence for the relationship between periodontitis and Alzheimer’s disease: A bidirectional Mendelian randomization study
Source: PLoS One. 2020 Jan 24;15(1):e0228206. doi: 10.1371/journal.pone.0228206 (PMC6980529; doi:10.1371/journal.pone.0228206)
Supplement: S1 Table — (DOCX) [file pone.0228206.s001.docx]

**S1 Table.** **Summary statistics for Mendelian randomization analysis of potential causal effect of periodontitis on Alzheimer’s disease**

| SNP | Chr | Nearest genes/contig | Alleles: effect^*^/other | Eaf^†^ | Exposure: chronic periodontitis | | | Outcome: AD [1] (21,982 cases vs. 41,944 controls) | | |
| --- | --- | --- | --- | --- | --- | --- | --- | --- | --- | --- |
|  |  |  |  |  | Coefficient^#^ | SE | *P* value | Coefficient^#^ | SE | *P* value |
| Munz *et al.* [2] (periodontitis 4,924 cases vs. 7,301 controls) | | | | | | | | | | |
| rs2064712 | 6 | *AL109933.3-AL391361.2* | A/G | 0.10 | 0.2136 | 0.0426 | 5.29E-07 | 0.0178 | 0.0199 | 0.3704 |
| rs16870060^¥^ | 8 | *MTND1P5* | G/T | 0.91 | 0.3070 | 0.0521 | 3.69E-09 | 0.0405 | 0.0259 | 0.1177 |
| rs729876 | 16 | *LOC107984137* | T/C | 0.90 | 0.2177 | 0.0380 | 9.77E-09 | 0.0049 | 0.0181 | 0.7845 |
| rs11084095 | 19 | *SIGLEC5* | A/G | 0.22 | 0.1503 | 0.0290 | 2.28E-07 | 0.0324 | 0.0147 | 0.0273 |
| rs9982623 | 21 | *MCM3AP* | C/T | 0.87 | 0.2135 | 0.0427 | 5.67E-07 | 0.0085 | 0.0224 | 0.704 |
| Shungin *et al.* [3] (periodontitis 12,289 cases vs. 22,326 controls) | | | | | | | | | | |
| rs13005050 | 2 | *AC139712.1* | C/T | 0.14 | 0.1432 | 0.031 | 3.76E-06 | -0.0242 | 0.0241 | 0.3153 |
| rs4956201 | 4 | *RPL34-AS1* | C/A | 0.89 | 0.2406 | 0.0474 | 3.89E-07 | 0.0196 | 0.0318 | 0.5368 |
| rs6816769 | 4 | *AC093867.3* | C/T | 0.89 | 0.1348 | 0.0294 | 4.57E-06 | -0.0342 | 0.0234 | 0.1428 |
| rs78422482 | 4 | *AC079755.6* | A/G | 0.01 | 0.2425 | 0.0510 | 2.02E-06 | 0.0303 | 0.0443 | 0.4935 |
| rs73155039 | 7 | *AC009541.16* | A/G | 0.99 | 0.8316 | 0.1757 | 2.22E-06 | 0.0144 | 0.0873 | 0.8687 |
| rs2976950 | 8 | *AC103957.8* | A/G | 0.60 | 0.0963 | 0.0195 | 7.99E-07 | -0.0043 | 0.0148 | 0.7715 |
| rs151226594 | 11 | *AP005273.3* | G/T | 0.01 | 0.3671 | 0.0768 | 1.75E-06 | -0.0338 | 0.0592 | 0.5682 |

AD: Alzheimer’s disease; Chr: chromosome; Eaf: effect allele frequency; SE: standard error; SNP: single-nucleotide polymorphism

^*^Effect allele carrier has increased risk of periodontitis; ^†^Frequency in 1000 Genomes Phase 3 combined population (<http://www.ensembl.org/index.html>); ^#^Coefficient: ln (odds ratio)

Five/seven SNPs in two GWAS were used as instrumental variables for chronic periodontitis respectively [2, 3]. Summary statistics of periodontitis GWAS of Munz *et al.* [2] were from Table 1 [pooled (no NL)] and Supplementary Table 3. Summary statistics of periodontitis GWAS for people with European ancestry excluding Hispanic/Latino background in the study of Shungin *et al.* [3] were downloaded from <https://data.bris.ac.uk/data/dataset/2j2rqgzedxlq02oqbb4vmycnc2>. Summary statistics of AD GWAS were from Stage 1 analysis of GWAS performed by Kunkle *et al.* [1], and the data was downloaded from <ftp://ftp.ebi.ac.uk/pub/databases/gwas/summary_statistics/KunkleBW_30820047_GCST007511>

^¥^rs16870054 (G/T) was used as proxy SNP (linkage disequilibrium R^2^>0.8) of rs16870060 (G/T) in the summary statistics of the outcome

**References**

1. Kunkle BW, Grenier-Boley B, Sims R, Bis JC, Damotte V, Naj AC, et al. Genetic meta-analysis of diagnosed Alzheimer's disease identifies new risk loci and implicates Abeta, tau, immunity and lipid processing. Nat Genet. 2019;51(3):414-30. Epub 2019/03/02. doi: 10.1038/s41588-019-0358-2. PubMed PMID: 30820047.

2. Munz M, Richter GM, Loos BG, Jepsen S, Divaris K, Offenbacher S, et al. Meta-analysis of genome-wide association studies of aggressive and chronic periodontitis identifies two novel risk loci. Eur J Hum Genet. 2019;27(1):102-13. Epub 2018/09/16. doi: 10.1038/s41431-018-0265-5. PubMed PMID: 30218097; PubMed Central PMCID: PMCPMC6303247.

3. Shungin D, Haworth S, Divaris K, Agler CS, Kamatani Y, Keun Lee M, et al. Genome-wide analysis of dental caries and periodontitis combining clinical and self-reported data. Nature communications. 2019;10(1):2773. Epub 2019/06/27. doi: 10.1038/s41467-019-10630-1. PubMed PMID: 31235808; PubMed Central PMCID: PMCPMC6591304.
