# Supplementary material for: Mixed evidence for the relationship between periodontitis and Alzheimer’s disease: A bidirectional Mendelian randomization study
Source: PLoS One. 2020 Jan 24;15(1):e0228206. doi: 10.1371/journal.pone.0228206 (PMC6980529; doi:10.1371/journal.pone.0228206)
Supplement: S2 Table — (DOCX) [file pone.0228206.s002.docx]

**S2 Table. Summary statistics for Mendelian randomization analysis of potential causal effect of Alzheimer’s disease on periodontitis**

| SNP | Chr | Nearest gene | Alleles: effect^*^/other | Eaf | Exposure: AD [1] (30,344 cases vs. 52,427 controls) | | | Outcome: periodontitis [2] (12,289 cases vs. 22,326 controls) | | |
| --- | --- | --- | --- | --- | --- | --- | --- | --- | --- | --- |
|  |  |  |  |  | Coefficient^#^ | SE | *P* value | Coefficient^#^ | SE | *P* value |
| rs4844610 | 1 | *CR1* | A/C | 0.19 | 0.1570 | 0.0175 | 3.60E-24 | 0.0154 | 0.0229 | 0.5012 |
| rs6733839 | 2 | *BIN1* | T/C | 0.41 | 0.1823 | 0.0128 | 2.10E-44 | -0.0003 | 0.0197 | 0.9871 |
| rs10933431 | 2 | *INPP5D* | C/G | 0.78 | 0.0943 | 0.0168 | 3.40E-09 | 0.0397 | 0.0247 | 0.1081 |
| rs9271058^¥^ | 6 | *HLA-DRB1* | A/T | 0.27 | 0.0953 | 0.0139 | 1.40E-11 | -0.0314 | 0.0405 | 0.4384 |
| rs9473117 | 6 | *CD2AP* | C/A | 0.28 | 0.0862 | 0.0141 | 1.20E-10 | -0.0178 | 0.0199 | 0.3709 |
| rs12539172 | 7 | *NYAP1* | C/T | 0.70 | 0.0834 | 0.0138 | 9.30E-10 | 0.0242 | 0.0194 | 0.2115 |
| rs10808026 | 7 | *EPHA1* | C/A | 0.80 | 0.1054 | 0.0141 | 1.30E-10 | 0.022 | 0.0222 | 0.3216 |
| rs73223431 | 8 | *PTK2B* | T/C | 0.37 | 0.0953 | 0.0139 | 6.30E-14 | -0.0346 | 0.0184 | 0.0609 |
| rs9331896 | 8 | *CLU* | T/C | 0.61 | 0.1278 | 0.0146 | 4.60E-24 | 0.0184 | 0.0183 | 0.3156 |
| rs7920721 | 10 | *ECHDC3* | G/A | 0.39 | 0.0770 | 0.0142 | 2.30E-09 | -0.0172 | 0.0192 | 0.3724 |
| rs3740688 | 11 | *SPI1* | T/G | 0.55 | 0.0834 | 0.0139 | 5.40E-13 | 0.0138 | 0.018 | 0.4418 |
| rs7933202 | 11 | *MS4A2* | A/C | 0.61 | 0.1165 | 0.0143 | 1.90E-19 | 0.0001 | 0.0182 | 0.9967 |
| rs3851179 | 11 | *PICALM* | C/T | 0.64 | 0.1278 | 0.0116 | 6.00E-25 | 0.0229 | 0.0184 | 0.2137 |
| rs11218343 | 11 | *SORL1* | T/C | 0.96 | 0.2231 | 0.0319 | 2.90E-12 | 0.0837 | 0.0451 | 0.0637 |
| rs17125924 | 14 | *FERMT2* | G/A | 0.09 | 0.1310 | 0.0202 | 1.40E-09 | -0.0154 | 0.0305 | 0.6144 |
| rs12881735 | 14 | *SLC24A4* | T/C | 0.78 | 0.0834 | 0.0139 | 7.40E-09 | 0.0284 | 0.021 | 0.1761 |
| rs138190086 | 17 | *ACE* | A/G | 0.02 | 0.2776 | 0.0483 | 7.50E-09 | 0.0006 | 0.0753 | 0.9934 |
| rs3752246 | 19 | *ABCA7* | G/C | 0.18 | 0.1398 | 0.0156 | 3.10E-16 | -0.021 | 0.0237 | 0.3751 |
| rs429358 | 19 | *APOE* | C/T | 0.22 | 1.2000 | 0.0192 | 1.20E-881 | -0.0218 | 0.0263 | 0.4074 |
| rs6024870 | 20 | *CASS4* | G/A | 0.91 | 0.1278 | 0.0202 | 3.50E-08 | -0.0068 | 0.0336 | 0.8387 |

AD: Alzheimer’s disease; Chr: chromosome; Eaf: effect allele frequency; SE: standard error; SNP: single-nucleotide polymorphism

^*^Effect allele carrier has increased risk of Alzheimer’s disease; ^#^Coefficient: ln (odds ratio)

These 20 SNPs were used as instrumental variables for AD. Summary statistics (coefficients and SEs) of AD GWAS were calculated based on odds ratios and 95% confidence intervals from Table 1 (Overall Stage 1+Stage 2, n=82,771) in the study of Kunkle *et al.* [1]. Summary statistics of periodontitis GWAS for people with European ancestry excluding Hispanic/Latino background in the study of Shungin *et al.* [2] were downloaded from <https://data.bris.ac.uk/data/dataset/2j2rqgzedxlq02oqbb4vmycnc2>.

^¥^rs9270225(G/A) was used as proxy SNP (linkage disequilibrium R^2^>0.8) of rs9271058(A/T) in the summary statistics of the outcome

**References**

1. Kunkle BW, Grenier-Boley B, Sims R, Bis JC, Damotte V, Naj AC, et al. Genetic meta-analysis of diagnosed Alzheimer's disease identifies new risk loci and implicates Abeta, tau, immunity and lipid processing. Nat Genet. 2019;51(3):414-30. Epub 2019/03/02. doi: 10.1038/s41588-019-0358-2. PubMed PMID: 30820047.

2. Shungin D, Haworth S, Divaris K, Agler CS, Kamatani Y, Keun Lee M, et al. Genome-wide analysis of dental caries and periodontitis combining clinical and self-reported data. Nature communications. 2019;10(1):2773. Epub 2019/06/27. doi: 10.1038/s41467-019-10630-1. PubMed PMID: 31235808; PubMed Central PMCID: PMCPMC6591304.
