# Supplementary figures and images for: Mixed evidence for the relationship between periodontitis and Alzheimer’s disease: A bidirectional Mendelian randomization study
Source: PLoS One. 2020 Jan 24;15(1):e0228206. doi: 10.1371/journal.pone.0228206 (PMC6980529; doi:10.1371/journal.pone.0228206)

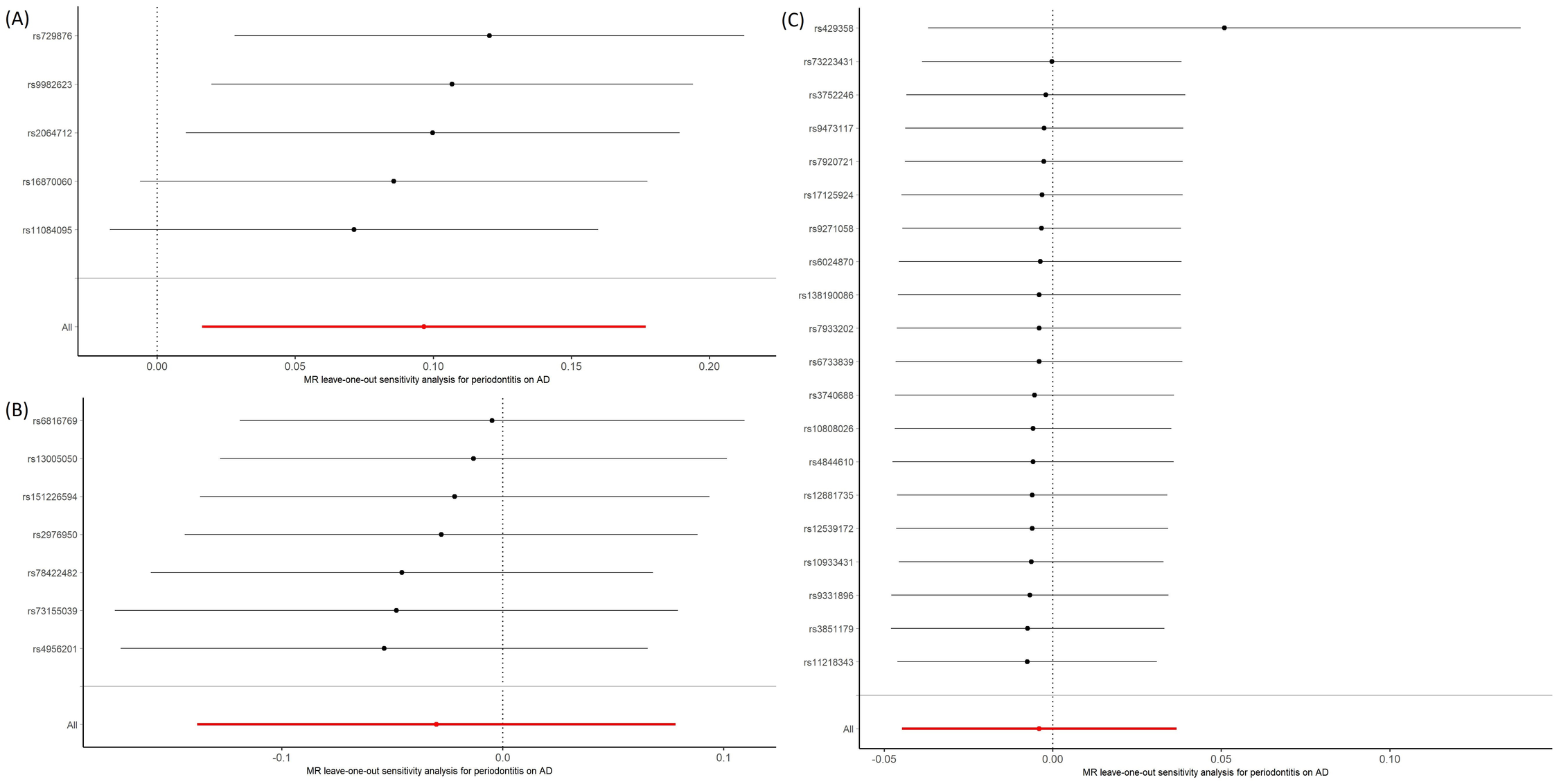

Supplement: S1 Fig — (A) Periodontitis on risk of Alzheimer’s disease using instrumental SNPs from Munz et al. (B) Periodontitis on risk of Alzheimer’s disease using instrumental SNPs from Shungin et al. (C) Alzheimer’s disease on risk of periodontitis. MR estimates were calculated using the inverse-variance weighted (IVW) method in a random effects model after excluding each individual single-nucleotide polymorphism (SNP). The scale on x-axis represents ln [odds ratio (OR)] for the risk of the outcome per genetically determined 1-unit increase in ln (OR) of the exposure. The dots represent the MR estimates and the lines represent 95% confidence interval of the estimates. AD: Alzheimer’s disease; MR: Mendelian randomization. (TIF) [file pone.0228206.s003.tif]
