# Supplementary material for: Mixed evidence for the relationship between periodontitis and Alzheimer’s disease: A bidirectional Mendelian randomization study
Source: PLoS One. 2020 Jan 24;15(1):e0228206. doi: 10.1371/journal.pone.0228206 (PMC6980529; doi:10.1371/journal.pone.0228206)
Supplement: Attachment — Submitted filename: Response_to_Reviewers_YQS_v2.0.docx [file pone.0228206.s007.docx]

**Relationship between periodontitis and Alzheimer’s disease: A bidirectional Mendelian randomization study** by Sun *et al.*

Manuscript ID: PONE-D-19-17973

**Response to the reviewers**

We would like to express our sincere thanks to the reviewers for their time and comments. Replies to points are indicated by text in blue color, and changes to the paper as a result of these comments are clearly indicated.

After we submitted the manuscript to PLOS ONE in June, a new genome-wide association study (GWAS) of periodontitis [1] was published, and the summary statistics of one of the latest GWAS of Alzheimer’s disease (AD) [2] became publicly available. We feel we are obliged to update the analyses in our study with the latest data to avoid drawing an incorrect conclusion for this important research question. For the updated analyses, we used summary statistics of the study by Kunkle *et al.* [2] instead of the study by Jansen *et al.* [3] for AD. This is because the former study used clinically diagnosed AD as cases and the latter study included AD-by-proxy phenotype as cases. More accurate definition of the trait should lead to less bias in the results. We also used instrumental SNPs from the new GWAS of periodontitis [1] to validate our results using SNPs from the study by Munz *et al.* [4]. There is no overlap between the instrumental SNPs in the two GWAS [1, 4].

Based on the latest data and the reviewers’ comments, we revised the manuscript accordingly and responded point by point to the comments. We believe that the manuscript has been largely improved and hope it is qualified for publication at PLOS ONE.

Reviewers’ comments:

**Reviewer #1:**

I recommend resubmitting with major revisions. First, the thoroughness of the analysis is commendable. The use of multiple mendelian randomization (MR) estimate methods, pleiotropy outlier measurements, reverse-causality, and leave-one-out analysis provide a better understanding of the results. However, the clumping threshold of the SNPs tested should be reported. This is to minimize the impact of linkage disequilibrium.

Reply: We had performed the clumping of the instrumental SNPs with clumping R^2^ cut-off 0.001. The description has now been added into the “Materials and methods” section.

Unfortunately, the weakest parts of the manuscript are the data used for the analysis. First, the exposure data, the GWAS of periodontitis, only included five SNPs with relatively high P value threshold (5e-6). The norm is to use P value of 5e-8 and include more SNPs. I recommend calculating and reporting the F-statistic and the R2 value, which should explain the strength of the instrument and the variance of the SNPs. It would help assert the first assumption of MR: the instrumental variance is robustly associated with the exposure. The authors somewhat address this in the discussion ("…bias from weak instruments shifts the MR estimate towards the direction of the null…") but quantifying the instrument strength would put the results into greater context.

Reply: In addition to using the 5 SNPs from the study by Munz *et al.* [4] as instruments for periodontitis, we now have used a new set of instrumental SNPs (7 SNPs) from a lately published GWAS of periodontitis [1] to validate the findings. We had chosen a less stringent *P* value (5e-6) instead of the standard 5e-8 in order to include more SNPs as instruments. When using the standard *P* value cut-off (5e-8), only 2 and 1 SNPs remained significant in the two GWAS of periodontitis respectively [1, 4].

We have calculated F-statistic and the R^2^ value for the instruments and added them in the first paragraph of the “Results” section. We need to emphasize that the strength of the instruments was strong for Alzheimer’s disease but weak for periodontitis, as indicated by the F-statistics and the R^2^ values. More discussion about the weak instruments for periodontitis has been added in the revised manuscript.

Similarly, the small GWAS study used for the reverse causation analysis (2,681 cases and 1,823 controls) may not provide sufficient power. As such, it is unclear that the result of the reverse causation analysis is due to lack of reverse causality or from lack of statistical power. The authors should provide the appropriate statistical power calculations to address this possibility.

Reply: We have now used the latest and largest GWAS of periodontitis consisting 12,289 clinically diagnosed cases vs. 22,326 controls [1] for the reverse causation analysis. The *post hoc* power calculations may not be appropriate when the analyses have already been performed [5-7]. However, confidence intervals (CIs) obtained can give an insight into power [5-7]. In particular, narrow 95% CIs of the estimates (Table 1 in the revised manuscript) suggest that the analysis had sufficient power to investigate a causal effect of liability of AD on risk of periodontitis.

Lastly, the outcome data, the GWAS of Alzheimers Disease (AD), is not the latest GWAS of the given disease and does not include the latest loci associated with AD. Latest meta-analysis GWAS has found 29 loci associated with AD (Jansen et al. 2019), while this manuscript refers to only nineteen loci found in an older GWAS. The summary statistics from the latest study are publicly available. The authors should run the analyses again, both forward and reverse causation analyses, using this dataset as their AD data.

Reply: Now we have used summary statistics from one [2] of the latest and largest GWAS of AD [2, 3] to run the bidirectional MR analyses. The results have been updated in the revised manuscript. The reason why we used data from Kunkle *et al.* instead of from Jansen *et al.* was explained in the first part of this reply letter. We hope the reviewer agrees with our choice.

In summary, I recommend major revision of the manuscript. Clumping threshold should be reported. F-statistics and R2 of all exposure instruments, as well as the power calculation for the reverse causation analysis should be calculated and reported. Finally, additional analysis using a more up-to-date AD GWAS summary data should be done.

Reply: Please see replies to points as above.

**Reviewer #2:**

Summary:

Mendelian randomization (MR) is a strong tool for a better understanding of potential causal effects especially in disorders with a long-term duration such as AD. However, if not done properly, MR studies are prone to misinterpretation due to the complexities involved in the analysis. In this paper, the authors have looked at the relationship between AD and periodontitis. They have used summary stats from the related GWAS studies and concluded that there is no relationship. The authors have performed extensive analysis utilizing a number of MR techniques.

Reply: We have now updated the analyses using the latest summary statistics that became available after we had submitted our manuscript in June 2019.

Issues:

- First, the analysis is not public. In order to ensure reproducibility, it is crucial to make the code or analysis notebook publicly available. Especially with computational and data analysis work, unintentional coding mistakes might be made; availability of code/notebook helps with the review and future reproducibility.

Reply: We have now made R code as well as the data used in the R code available as S2 and S3 Files. The R code is for performing MR of periodontitis on the risk of Alzheimer’s using 5 SNPs from the study of Munz *et al* [4]*.* The other MR analyses in the study used similar code and the data can be extracted from S1 and S2 Tables.

- Another major issue with the paper is not using the latest Alzheimer GWAS. In the latest study, 29 risk-loci were identified https://www.nature.com/articles/s41588-018-0311-9

Reply: Now we have used summary statistics from one [2] of the latest and largest GWAS of AD [2, 3]. The results have been updated in the revised manuscript. The reason why we used data from Kunkle *et al.* instead of from Jansen *et al.* was explained in the first part of the reply letter.

- Periodontitis SNPs are limited and very strong. Using a more relaxed p-value helps, 5e-8. Better to use R^2 for choosing the instrumental variable.

Reply: In addition to using the 5 SNPs from the study by Munz *et al.* [4] as periodontitis instruments, we have now used a new set of instrumental SNPs (7 SNPs) for periodontitis in a lately published GWAS [1] to validate the findings. We had chosen to use a less stringent *P* value (5e-6) instead of the standard 5e-8 in order to include more SNPs as instruments. When using the standard *P* value cut-off (5e-8), only 2 and 1 SNPs remained significant in the two GWAS of periodontitis respectively [1, 4].

We have calculated F-statistic and the R^2^ value for the instruments and added them in the first paragraph of the “Results” section. We need to emphasize that the strength of the instruments was strong for Alzheimer’s disease but weak for periodontitis, as indicated by the F-statistics and the R^2^ values. More discussion about the weak instruments for periodontitis has been added in the revised manuscript.

- MR analysis: authors have used multiple methods; however, some details are not properly reported such as SNPs clumping threshold.

Reply: We had performed the clumping of the instrumental SNPs with a clumping R^2^ cut-off 0.001. Now the description has been added into the “Materials and methods” section.

**References:**

1.	Shungin D, Haworth S, Divaris K, Agler CS, Kamatani Y, Keun Lee M, et al. Genome-wide analysis of dental caries and periodontitis combining clinical and self-reported data. Nature communications. 2019;10(1):2773. Epub 2019/06/27. doi: 10.1038/s41467-019-10630-1. PubMed PMID: 31235808; PubMed Central PMCID: PMCPMC6591304.

2.	Kunkle BW, Grenier-Boley B, Sims R, Bis JC, Damotte V, Naj AC, et al. Genetic meta-analysis of diagnosed Alzheimer's disease identifies new risk loci and implicates Abeta, tau, immunity and lipid processing. Nat Genet. 2019;51(3):414-30. Epub 2019/03/02. doi: 10.1038/s41588-019-0358-2. PubMed PMID: 30820047.

3.	Jansen IE, Savage JE, Watanabe K, Bryois J, Williams DM, Steinberg S, et al. Genome-wide meta-analysis identifies new loci and functional pathways influencing Alzheimer's disease risk. Nat Genet. 2019;51(3):404-13. Epub 2019/01/09. doi: 10.1038/s41588-018-0311-9. PubMed PMID: 30617256.

4.	Munz M, Richter GM, Loos BG, Jepsen S, Divaris K, Offenbacher S, et al. Meta-analysis of genome-wide association studies of aggressive and chronic periodontitis identifies two novel risk loci. Eur J Hum Genet. 2019;27(1):102-13. Epub 2018/09/16. doi: 10.1038/s41431-018-0265-5. PubMed PMID: 30218097; PubMed Central PMCID: PMCPMC6303247.

5.	Hoenig JM, Heisey DM. The abuse of power: the pervasive fallacy of power calculations for data analysis. . Am Stat. 2001;55(1):19-24.

6.	Levine M, Ensom MH. Post hoc power analysis: an idea whose time has passed? Pharmacotherapy. 2001;21(4):405-9. Epub 2001/04/20. PubMed PMID: 11310512.

7.	Walters SJ. Consultants' forum: should post hoc sample size calculations be done? Pharm Stat. 2009;8(2):163-9. Epub 2008/04/18. doi: 10.1002/pst.334. PubMed PMID: 18416448.
