# Supplementary material for: Mixed evidence for the relationship between periodontitis and Alzheimer’s disease: A bidirectional Mendelian randomization study
Source: PLoS One. 2020 Jan 24;15(1):e0228206. doi: 10.1371/journal.pone.0228206 (PMC6980529; doi:10.1371/journal.pone.0228206)
Supplement: Attachment — Submitted filename: Response_to_Reviewers_YQS_R1.docx [file pone.0228206.s008.docx]

**Relationship between periodontitis and Alzheimer’s disease: A bidirectional Mendelian randomization study** by Sun *et al.*

Manuscript ID: PONE-D-19-17973R1

**Response to the reviewers**

We are glad the reviewers are satisfied with our revision R1. Replies to further points raised by Reviewer #1 are indicated by text in blue color. Based on Reviewer #1’s comments, we changed the title of the manuscript accordingly. We hope the manuscript is now qualified for publication at PLOS ONE.

Reviewers’ comments:

**Reviewer #1:**

I recommend resubmitting with minor revisions. I would like to thank the authors for the additional transparency, including the inclusion of F-statistics, clumping threshold, and the analysis code. While the periodontitis data, including the new data from Shungin et al., have fairly weak instrumental strength, quantifications of the instrumental strengths contextualize the mixed, and likely null, results. Additions to the Discussion and the Conclusion further contextualize and underline the limitations of the manuscript and what would be necessary for more robust future analyses. The title should echo this change and could be updated to reflect the null or mixed evidence for the relationship between periodontitis and Alzheimers Disease (AD).

I still believe that including AD GWAS with proxy cases (Janssen et al.) would be ideal but not necessary. AD has a strong heritability and the potential for introducing additional bias through the inclusion of proxy cases is minimal. However, larger GWAS size for AD will not solve the main issue of the analysis, which is the weak periodontitis data.

In summary, I recommend minor revision of the manuscript. Title should be changed to reflect the mixed/null finding of the manuscript. While not essential, I also recommend additional analysis using AD GWAS data with proxy cases.

Reply: We have changed the title to “Mixed evidence for the relationship between periodontitis and Alzheimer’s disease: A bidirectional Mendelian randomization study”.

We have performed the additional MR analyses of periodontitis on risk of Alzheimer’s disease (AD), using data of AD GWAS by Jansen *et al.* that included AD-by-proxy phenotype as AD cases. Since less accurate definition of the trait (misclassification of Alzheimer’s disease) likely leads to bias in the MR results, and the main conclusion did not change, we present the results here (Table and Figure as below) and did not include the additional analyses in the revised manuscript.

**Table. MR estimates for the association between chronic periodontitis and Alzheimer’s disease**^†^

| Method | Periodontitis on Alzheimer’s disease | | | | | |
| --- | --- | --- | --- | --- | --- | --- |
|  | Instrumental SNPs^*^ from Munz *et al.* | | | Instrumental SNPs^#^ from Shungin *et al.* | | |
|  | OR (95% CI) | *P* value | Q statistic/ *P* value | OR (95% CI) | *P* value | Q statistic/ *P* value |
| IVW | 1.00 (0.98, 1.02) | 0.95 | 11.85/0.02 | 0.99 (0.97, 1.01) | 0.23 | 5.19/0.64 |
| Weighted median | 1.00 (0.98, 1.02) | 0.86 |  | 0.99 (0.96, 1.01) | 0.17 |  |
| MR-Egger | 0.93 (0.88, 0.98) | 0.07 | 3.16/0.37 | 0.99 (0.97, 1.03) | 0.80 | 5.07/0.53 |
| MR-RAPS | 1.00 (0.98, 1.02) | 0.83 |  | 0.99 (0.97, 1.01) | 0.22 |  |

CI: confidence interval; OR: odds ratio; IVW: inverse-variance weighted; MR: Mendelian randomization; RAPS: robust adjusted profile score; SNP: single-nucleotide polymorphism

^†^Summary statistics of Alzheimer’s disease were from Phase 3 of GWAS by Jansen *et al.* (71,880 cases including AD-by-proxy phenotype vs. 383,378 controls); ^*^Five/eight^#^ SNPs were used as instrumental variables for periodontitis respectively

**Figure. Mendelian randomization (MR) estimates for the risk of Alzheimer’s disease associated with periodontitis.**

(A) Using instrumental SNPs from Munz *et al.* for periodontitis. (B) Using instrumental SNPs from Shungin *et al.* for periodontitis. Summary statistics of Alzheimer’s disease were from Phase 3 of GWAS by Jansen *et al.* (71,880 cases including AD-by-proxy phenotype vs. 383,378 controls). MR estimates were calculated using the inverse-variance weighted (IVW) method to summarize the effect from each individual single-nucleotide polymorphism (SNP) in a random effects model. Odds ratio (OR) represents the risk of Alzheimer’s disease per genetically determined 1-unit increase in ln (OR) of periodontitis. 95% CI: 95% confidence interval of the odds ratio. AD: Alzheimer’s disease; IV: instrumental variable

**Reviewer #2:**

Authors have addressed all the issues adequately. The availability of the analysis code will be helpful for future replication.
